# Full antagonist of the IL-7 receptor suppresses chronic inflammation in non-human primate models by controlling antigen-specific memory T cells

**DOI:** 10.15698/cst2018.12.168

**Published:** 2018-12-10

**Authors:** Lyssia Belarif, Bernard Vanhove, Nicolas Poirier

**Affiliations:** 1 OSE Immunotherapeutics, Nantes, France.

**Keywords:** Chronic inflammation, IL-7, IL-7 receptor, memory T cells, monoclonal antibody

## Abstract

Targeting the expansion of pathogenic memory immune cells is a promising therapeutic strategy to prevent chronic autoimmune attacks. Interleukin 7 (IL-7) is a limiting and potent cytokine produced by epithelial and stromal cells sustaining T-lymphocytes development, homeostasis and cell metabolism. Almost all conventional mature T lymphocytes express the IL-7 receptor (IL-7R), with the exception for naturally-occurring regulatory T-cells (Treg), constituting a rare opportunity to selectively strangle pathogenic effectors while preserving crucial natural regulators. In our recent study, we reported that therapeutic efficacy of antagonist anti- IL-7Rα mAbs in a non-human primate model of memory T cell-induced chronic inflammation depends on recognition of an epitope overlapping the IL-7 binding domain (site 1) and the receptor heterodimerization region (site-2b) (Nat Commun, 9(1):4483). We found that “site-1-only” mAbs prevented IL-7-induced JAK/STAT signaling but induced PI3K and Erk signaling and lacked efficacy *in vivo*, whereas “site-1 + 2b” mAbs were fully antagonist and demonstrated potent activity to control skin inflammation on the long term. The mechanism of action comprised the neutralization of IFN-γ producing antigen-specific memory T cells, without inducing lymphopenia or polyclonal T-cell functional or metabolic defects as generally observed previously in rodents.

Targeting proinflammatory cytokines such as TNFα, IL-1, IL-6, is clinically beneficial in several immune-mediated inflammatory diseases (IMID). However, these cytokines are downstream mediators of immune responses that show poor impact on upstream drivers of dysregulated immune responses. Thus, by addressing consequences and not sources of immune disorders, drugs that target cytokines are not effective in all patients or diseases, depending of etiologies, and significant rates of primary and secondary resistances are still observed. Obviously, changing our approach to treat IMIDs by targeting the fuel of immune responses might prevent pathogenic cell development, survival and accumulation, and therefore might tackle relapses and synergize with conventional and anti-proinflammatory cytokine-targeting drugs.

Interleukin 7 (IL-7) is a non-classical cytokine, since it is produced essentially by non-hematopoietic stromal cells and acting vitally on the majority of immune cells. IL-7 is produced by epithelial and mesenchymal cells in lymphopoietic tissues such as bone marrow, thymus, spleen, lymph nodes and gut. Furthermore, several cell types such as keratinocytes, hepatocytes, lymphatic endothelial cells and articular chondrocytes have been also described to produce IL-7. In contrast, immune cells such as T cells, B cells and natural killer (NK) cells do not produce IL‑7, although small amounts of IL-7 could be produced by some subsets of dendritic cells. Almost all conventional mature T lymphocytes express the IL-7 receptor (IL-7R), with the exception of naturally-occurring regulatory T-cells (Treg), constituting a rare opportunity to selectively strangle pathogenic effectors while preserving crucial natural regulators. Based on this concept, many preclinical studies in rodents have highlighted that targeting the IL-7/IL-7R pathway holds promise for the treatment of IMID or to prevent transplant rejection and has the potential to restore immune tolerance. Dysregulated IL-7/IL-7R signaling and numerous increased risk association with IL-7R polymorphisms have been observed in patients suffering from IMID (e.g. multiple sclerosis, type 1 diabetes, rheumatoid arthritis, inflammatory bowel diseases).

IL-7 signals through the cell surface IL-7 receptor (IL-7R) that consists of a specific IL-7R alpha chain (IL-7Rα, CD127) which dimerizes with the common cytokine receptor gamma chain (γc, CD132). IL-7 interacts with both domain D1 of the IL-7Rα (site-1) and domain D1 of the γ-chain subunit (site-2a); IL-7Rα and the γ-chain also interact together with their D2 domains (site-2b), stabilizing and forming an active IL-7/IL-7Rα/γ-chain ternary complex upon activation (**Figure 1**). IL-7R delivers proliferative and anti-apoptotic signals by activating phosphoinositide 3‑kinase (PI3K), extracellular signal-regulated kinase (ERK) and Janus kinase-signal transducer and activator of transcription (JAK-STAT) pathways as well as regulating expression of anti- and pro-apoptotic BCL-2 family members.

**Figure 1:**
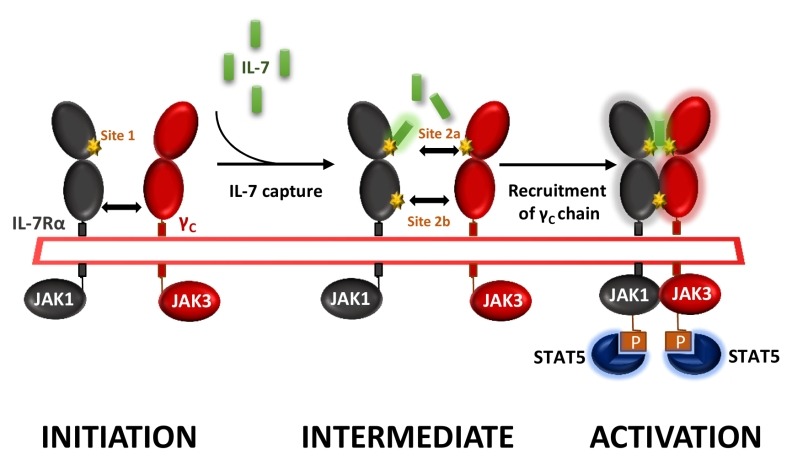
FIGURE 1: Cytokine-induced receptor heterodimerization signaling mechanisms. During the initiation step, IL-7 interacts with the extracellular domain 1 (D1) of IL-7Rα, generating the site-1 interface. This leads to the intermediate step where a 1:1 complex can associate with the shared common gamma-chain (γ_c_) receptor. The binding of γ_c_ receptor involves an interface between IL-7 and γ_c_ called site-2a and an interface between D2 regions of the IL-7Rα and γ_c_ receptor called site-2b. The stabilized heterodimer complex activates the JAK/STAT and possible other signaling pathways.

We generated and screened new anti-human IL-7Rα mAbs for their ability to antagonize IL-7-induced STAT5 phosphorylation on human peripheral T lymphocytes in vitro. Epitope characterization identified two types of anti-human IL-7Rα antagonist mAbs: (1) mAbs binding to the region (site-1) of interaction with IL-7, as previously reported by others, and (2) a mAb which binds an epitope overlapping both site-1 and the domain (site-2b) of heterodimerization between IL-7Rα and the γ-chain subunits (**Figure 1**). In this study we were intrigued by the lack of efficacy of site-1 mAbs, as compared with site-1/2b mAbs, to control memory T-cells-induced skin inflammation and T-cell dependent humoral response in cynomolgus monkey, despite presenting similar pharmacological antagonist properties *in vitro* and pharmacokinetic parameters *in vivo*. Similar lack of efficacy with a site-1 only mAb has been previously reported in an experimental autoimmune encephalomyelitis (EAE) monkey model. We found that IL-7 antagonist mAbs targeting at site-1 only induced receptor internalization, as previously reported by others, whereas site-1/2b mAb prevented internalization. Coming back to *in vitro* characterization of these mAbs, we found that all site-1 mAbs induce some level of ERK and PI3K signaling, even in the absence of IL-7, while the site-1/2b remained a strict antagonist. RNA-sequencing of human leukocytes incubated with different mAbs revealed that site-1 only mAbs, but not site-1/2b mAb, induced significant transcriptomic modification related to leukocytes activation, differentiation, proliferation and inflammatory responses associated with the MAPK/ERK pathway confirming the dual agonist/antagonist properties of site-1 only mAbs.

IL-7Rα deficiency or mutation in mice and humans lead to a broad deficiency in T cell-development. Similarly, in most previous preclinical studies performed in rodents, blocking IL-7R induced broad lymphodepletion and potent immunosuppression. However, in our study we observed that administration of high doses of mAbs, either targeting at site-1 or site1/2b, did not significantly change leukocytes cell counts, the frequency of T cell subsets in periphery or their *ex vivo* metabolism. Previous reports by others also described that anti-IL-7Rα injection at high dose in marmo-set or cynomolgus monkey did not result in lymphodepletion either. Similarly, lymphopenia has not been reported in recent phase I clinical trials with two different site-1 anti-IL-7Rα in healthy volunteers or in type 1 diabetic adult patients. Finally, we found that the efficacy of a single administration of the site-1/2b mAb to control skin inflammation in vivo is long-lasting since - despite monthly chronic antigen challenges - animals remained protected after complete drug elimination and inflammation did not recover over as long as a year. These animals were otherwise immunocompetent and able to mount new immune responses. However, *ex vivo*, memory T cells response against antigens administered together with site-1/2b mAb was abrogated. These data are compatible with our observations that IL-7 has poor effect on polyclonal stimulation of naïve T cells, while apoptosis and proliferation of antigen restimulated human memory T cells is tightly controlled by IL-7.

**Figure 2:**
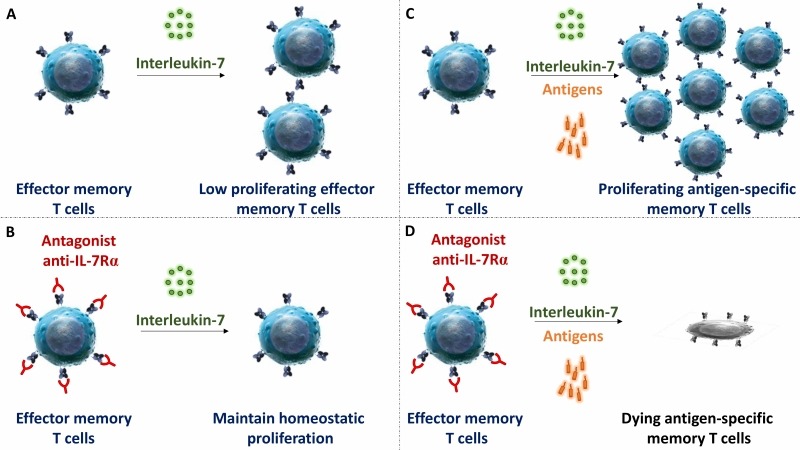
FIGURE 2: Blockade of IL-7R selectively controls survival and expansion of stimulated antigen-specific memory T cells. No antigenic stimulation: Interleukin-7 was described to control some degree of lymphocyte homeostasis **(A)**; however blockade of IL7R in human and non-human primates does not modify the peripheral pool of T cells **(B)**. Antigen restimulation: IL-7 is crucial to prevent apoptosis and sustain memory T cells proliferation after antigen restimulation **(C)**. Full antagonistic anti-IL7R mAb blunts selectively antigen-specific memory T cells responses **(D)**.

Primary and acquired resistance to current drugs in autoimmune and chronic inflammatory diseases as well as recurrence of chronic inflammation is governed by dysregulated re-activation and proliferation of pathogenic memory T cells. In this study, we reported that a strict antagonist of IL-7Rα prevents on the long-term memory T cell mediated skin inflammation in primates, without induction of lymphopenia or polyclonal T cell functional or metabolic deficiencies. Blocking IL-7Rα during antigen recall selectively abrogated the response of antigen-specific memory T lymphocytes without impacting homeostasis and response of quiescent T lymphocytes (**Figure 2**).

